# Editorial: Cancer diagnostics in solid tumors-from pathology to precision oncology

**DOI:** 10.3389/fmolb.2023.1150641

**Published:** 2023-02-21

**Authors:** Umberto Malapelle, Pedro Borralho, Liang Wang, Fernando Schmitt

**Affiliations:** ^1^ Department of Public Health, University of Naples Federico II, Naples, Italy; ^2^ Novartis Farma - Produtos Farmacêuticos, Porto Salvo, Portugal; ^3^ Department of Tumor Biology, Moffitt Cancer Center & Research Institute Tampa, Tampa, FL, United States; ^4^ Department of Pathology, Faculty of Medicine of University of Porto, Porto, Portugal

**Keywords:** precision oncology, molecular pathology, tumors, molecular biology, pathologist

In the novel era of target treatments for advanced stage cancer patients, personalized medicine is a rapidly evolving field. This phenomenon led to a paradigm shift in disease diagnosis and treatment, from the traditional patient stratification based on phenotypic biomarkers to ever-increasing customization and tailoring of diagnostic and therapeutic interventions. In this scenario, molecular testing on biological specimens, including tissue and liquid biopsies, plays a key role in the assessment of clinically relevant biomarkers status with diagnostic, prognostic, and predictive values to tailor and track, therapeutic interventions, and outcomes along the full patient treatment continuum. (La Thangue and Kerr, 2011). In this setting, modern pathologists, who are long-standing gatekeepers of cancer diagnostics, should evolve with the development of a novel figure of molecular pathologist. (Pisapia et al., 2022). Currently, this figure plays a pivotal role in multidisciplinary teams and molecular tumor boards, bridging anatomic pathology with cellular and molecular biology, being increasingly involved in multidisciplinary treatment decisions and response monitoring, far beyond their traditional role in cancer diagnosis. (Pisapia et al., 2022).

Overall, in this Research Topic of Frontiers in Molecular Biosciences, we attempt to address the state of art and the future perspectives in the field of precision medicine and integrative pathology.

Regarding lung cancer, the adoption of novel molecular approaches enables the possibility to add more pathological, biomolecular, and clinical information to rare and non-codified clinical entities, with an increase in the knowledge and the expertise to manage this specific histotypes, as reported by Parente et al. Another interesting field of investigation is represented by micro-RNA (miRNA). As reported by Tiang et al., miR-125b-5p may be adopted as a prognostic marker and a therapeutic target for lung adenocarcinoma. (Tang et al.). Molecular biology may be used not only in advanced stage of disease but also to predict the prognosis of early-stage lung adenocarcinoma and guide the selection of adjuvant therapy for early-stage lung adenocarcinoma patients, as demonstrated by Zhao et al. (Zhao Y et al., 2022).

As far as central nervous system neoplasms are concerned, Zheng et al. highlighted that a 21 gene-pair signature based on relative expression ordering may be useful to identify high-risk low grade gliomas patients in order to guide timely intervention. (Zheng et al.). In addition, further advances have been made in the field of glioblastoma management with the identification of TSPAN4 expression as a prognostic and immune target and RCN1 as a potential targeted in these patients. (Lu et al.), (Zheng et al.).

Considering breast cancer, novel therapeutic strategies have been developed. In particular, triple negative breast cancer patients may benefit from immune-checkpoint inhibitors after PD-L1 evaluation, (Ribeiro et al.), whereas *PIK3CA* mutated advanced ER+/HER2-breast cancer patients can be treated with specific PIK3CA inhibitors (Peixoto et al.), with a significant improvement in clinical outcomes.

Interesting results have also been obtained in gastric cancer. In particular, Hong *et al* proposed an integrated analysis of prognostic mRNA signature in early- and progressive-stage gastric adenocarcinomas (Hong et al.).

Regarding hepatocellular carcinoma, Zhao et al. reported that ALG3 was overexpressed and can be considered a potential indicator of survival in these patients. (Zhao et al., 2022). Remarkably, as it has been reported by Roa-Colomo et al. the identification and characterization of circulating epithelial cells by ASGR1 and/or miR-122-5p expression may be used as a risk-stratification and independent prognostic tool in liver cirrhosis and early stage hepatocellular carcinoma patients. (Roa-Colomo et al.).

Considering pancreatic cancer, it has been demonstrated that a comprehensive analysis of Lysyl Oxidase family members, in particular LOX and LOXL2, might have a prognostic and predictive value in pancreatic adenocarcinoma. (Jiang et al.).

A novel prognostic stratification model based on neutrophil extracellular traps signature has been proposed by Chen et al. for Head and Neck squamous cell carcinoma. (Chen et al.).

As far as genital-urinary tract is concerned, important advances have been made. In particular, Xia et al. proposed a novel gene signature associated with “E2F target” to predict the prognosis in prostate cancer (Xia et al.). Considering kidney, a significant prognostic role was associated with mTORC1 signaling pathway-related genes and pyroptosis-related genes and tumor microenvironment infiltration (Zhang et al.; Zhanghuang et al.).

Beyond a specific cancer type, Wu et al. highlighted the oncogenic role of SNRPB expression. (Wu et al.).

Overall, this Research Topic has highlighted the role of molecular testing and of the novel figure of molecular pathologist in the management of cancer patients.

Considering the implementation of next-generation approaches, based on spatial transcriptomics and digital pathology, and in particular the possibility to simultaneously combine these data with the molecular status, the evolving field of modern Anatomic Pathology will be definitely revolutionized during the next years. In this setting, pathologists should take care not only of morphological aspects but also of the integrated data coming from different cancer types, also by using Artificial Intelligence based tools. (Bonizzi et al.; Souza da Silva et al.). Ongoing research is warranted to improve the clinical outcome of cancer patients.

## References

[B1] La Thangue N. B. Kerr D. J. (2011). Predictive biomarkers: A paradigm shift towards personalized cancer medicine. Nat. Rev. Clin. Oncol. 8, 587–596. 10.1038/nrclinonc.2011.121 21862978

[B2] Pisapia P. L'Imperio V. Galuppini F. Sajjadi E. Russo A. Cerbelli B. (2022). The evolving landscape of anatomic pathology. Crit. Rev. Oncol. Hematol. 178, 103776. 10.1016/j.critrevonc.2022.103776 35934262

[B3] Tang L. Yuan Y. Zhai H. Wang J. Zhang D. Liang H. (2022). MicroRNA-125b-5p correlates with prognosis and lung adenocarcinoma progression. Front. Mol. Biosci. 8, 788690. 10.3389/fmolb.2021.788690 35187068 PMC8851393

[B4] Zhao Y. Qing B. Xu C. Zhao J. Liao Y. Cui P. (2022). DNA damage response gene-based subtypes associated with clinical outcomes in early-stage lung adenocarcinoma. Front. Mol. Biosci. 9, 901829. 10.3389/fmolb.2022.901829 35813819 PMC9257065

[B5] Zheng Y. Lang Y. Qi B. Wang Y. Gao W. Li T. (2023). TSPAN4 is a prognostic and immune target in Glioblastoma multiforme. Front. Mol. Biosci. 9, 1030057. 10.3389/fmolb.2022.1030057 36685274 PMC9853066

